# High-quality permanent draft genome sequence of *Bradyrhizobium* sp. Ai1a-2; a microsymbiont of *Andira inermis* discovered in Costa Rica

**DOI:** 10.1186/s40793-015-0007-z

**Published:** 2015-06-14

**Authors:** Rui Tian, Matthew Parker, Rekha Seshadri, TBK Reddy, Victor Markowitz, Natalia Ivanova, Amrita Pati, Tanja Woyke, Mohammed Baeshen, Nabih Baeshen, Nikos Kyrpides, Wayne Reeve

**Affiliations:** 1 Centre for Rhizobium Studies, Murdoch University, Murdoch, Australia; 2 Binghamton University, State University of New York, New York, USA; 3 DOE Joint Genome Institute, Walnut Creek, California USA; 4 Biological Data Management and Technology Center, Lawrence Berkeley National Laboratory, Berkeley, California USA; 5 Center of Nanotechnology, King Abdulaziz University, Jeddah, Saudi Arabia; 6 Department of Biological Sciences, Faculty of Science, Jeddah University, Jeddah, Saudi Arabia; 7 Department of Biological Sciences, Faculty of Science, King Abdulaziz University, Jeddah, Saudi Arabia

**Keywords:** Root-nodule bacteria, Nitrogen fixation, Symbiosis, Alphaproteobacteria, GEBA-RNB

## Abstract

*Bradyrhizobium* sp. Ai1a-2 is is an aerobic, motile, Gram-negative, non-spore-forming rod that was isolated from an effective nitrogen fixing root nodule of *Andira inermis* collected from Tres Piedras in Costa Rica. In this report we describe, for the first time, the genome sequence information and annotation of this legume microsymbiont. The 9,029,266 bp genome has a GC content of 62.56% with 247 contigs arranged into 246 scaffolds. The assembled genome contains 8,482 protein-coding genes and 102 RNA-only encoding genes. This rhizobial genome was sequenced as part of the DOE Joint Genome Institute 2010 Genomic Encyclopedia for Bacteria and Archaea-Root Nodule Bacteria (GEBA-RNB) project proposal.

**Electronic supplementary material:**

The online version of this article (doi:10.1186/s40793-015-0007-z) contains supplementary material, which is available to authorized users.

## Introduction

*Bradyrhizobium* sp. strain Ai1a.2 is a representative of a distinctive lineage affiliated with the *Bradyrhizobium elkanii* superclade [1]. The *B. elkanii* superclade is one of the three main branches of the genus, together with the *B. japonicum*/*B. diazoefficiens* superclade [2,3], and the group encompassing photosynthetic *Aeschynomene* symbionts [4].

Members of the lineage represented by strain Ai1a.2 are readily diagnosed because they share a distinctive length variant in helix 9 within the 5′ intervening sequence region of the 23S rRNA gene [5]. Strain Ai1a.2 and its relatives have an insertion of 16 nucleotides in this region in comparison to *B. elkanii* USDA76, which can be identified by a straightforward PCR assay [6]. In a survey of 420 *Bradyrhizobium* strains from 25 countries [1], only 2% of the strains had this 23S rRNA length variant. These strains all clustered together into a strongly supported clade based on concatenated data for 23S rRNA and five protein-coding genes [1].

This clade was placed as the most basally diverging lineage within the *B. elkanii* superclade, and it included strains from three locations: Central America, the Caribbean, and South Africa. Strain Ai1a.2 was sampled in Costa Rica from the tree *Andira inermis* [6], and highly similar strains are also known to occur as symbionts of the same host legume in Panama [7]. Parker and Rousteau [8] also detected strains from this group in nodule samples from the beach legume *Canavalia rosea* in two Caribbean locations (Guadeloupe and Puerto Rico). Two *Bradyrhizobium* strains from distantly related legume hosts (*Leobordea* spp.) in South Africa (WSM2632, WSM2783) also belong to this clade [9].

*Andira inermis*, the host of strain Ai1a.2, is a large tree (up to 35 m height) commonly found in riparian habitats from southern Mexico through northern South America [10]. *Andira* was traditionally considered to be an early-diverging lineage within the Tribe Dalbergieae [11], but more recent phylogenetic analyses have suggested that it forms a separate lineage with unclear relationship to dalbergioid legumes [12]. Here we provide an analysis of the high-quality permanent draft genome sequence of *Bradyrhizobium* strain Ai1a.1. The fact that the genome of its close relative WSM2783 has also been sequenced as part of the Genomic Encyclopedia for Bacteria and Archaea-Root Nodule Bacteria (GEBA-RNB) project [13] will enable detailed comparative analysis of this group.

## Organism information

### Classification and features

**Figure 1:**
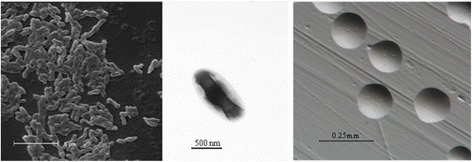
Images of *Bradyrhizobium* sp. Ai1a-2 using scanning (Left) and transmission (Center) electron microscopy as well as light microscopy to visualize colony morphology on solid media (Right).

*Bradyrhizobium* sp. Ai1a-2 is a motile, non-sporulating, non-encapsulated, Gram-negative strain in the order *Rhizobiales* of the class *Alphaproteobacteria*. The rod shaped form has dimensions of approximately 0.5 μm in width and 1.5-2.0 μm in length (Figure 1 Left and Center). It is relatively slow growing, forming colonies after 6–7 days when grown on half strength Lupin Agar (½LA) [14], tryptone-yeast extract agar (TY) [15] or a modified yeast-mannitol agar (YMA) [16] at 28°C. Colonies on ½LA are opaque, slightly domed and moderately mucoid with smooth margins (Figure 1 Right).

**Figure 2:**
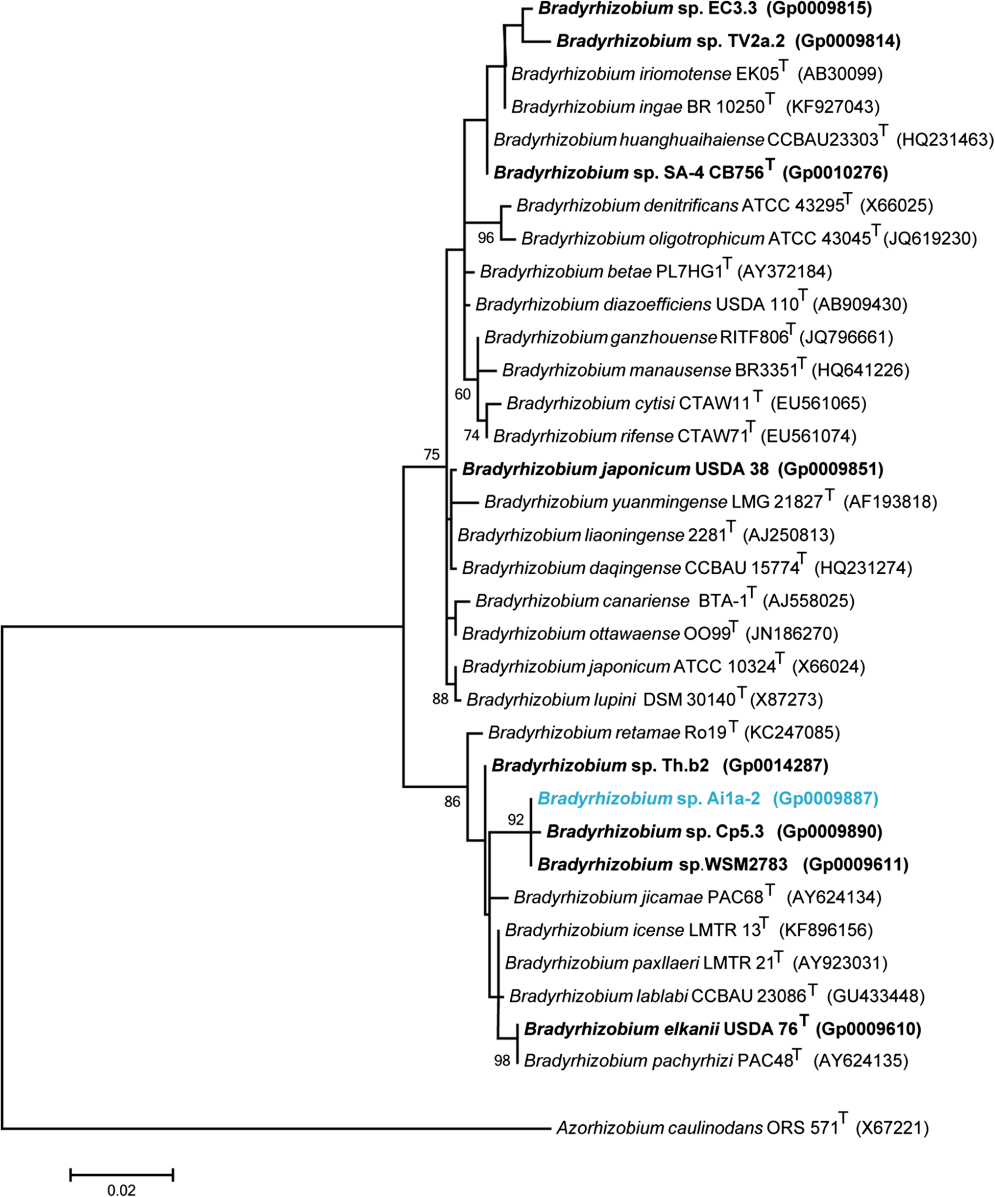
Phylogenetic tree showing the relationship of *Bradyrhizobium* sp. Ai1a-2 (shown in blue print) relative to other type and non-type strains in the *Bradyrhizobium* genus using a 1,310 bp intragenic sequence of the 16S rRNA gene. *Azorhizobium caulinodans* ORS 571^T^ sequence was used as an outgroup. All sites were informative and there were no gap-containing sites. Phylogenetic analyses were performed using MEGA, version 5.05 [37]. The tree was built using the maximum likelihood method with the General Time Reversible model. Bootstrap analysis with 500 replicates was performed to assess the support of the clusters. Type strains are indicated with a superscript T. Strains with a genome sequencing project registered in GOLD [20] have the GOLD ID mentioned after the strain number and are represented in bold, otherwise the NCBI accession number is provided.

Figure 2 shows the phylogenetic relationship of *Bradyrhizobium* sp. Ai1a-2 in a 16S rRNA gene sequence based tree. The 16S rRNA gene sequence of Aia1-2 (using a 1,370 bp intragenic sequence) is identical to that of *Bradyrhizobium* sp. WSM2783. *Bradyrhizobium* sp. Ai1a-2 is also closely related to *Bradyrhizobium* sp. Cp5.3 and *Bradyrhizobium* sp. Th.b2 with 16S rRNA gene sequence identities of 99.77% and 99.23%, respectively, as determined using NCBI BLAST analysis [17]. The highest identity (99.16%) of the 16S rRNA gene sequence of strain Ai1a-2 to type strain sequences occurs with *Bradyrhizobium icense* LMTR 13^T^ and *Bradyrhizobium paxllaeri* LMTR 21^T^ based on alignment using the EzTaxon-e server [18,19].

Minimum Information about the Genome Sequence (MIGS) is provided in Table 1 and Additional file 1: Table S1.

**Table 1:** **Classification and general features of** ***Bradyrhizobium*** **sp. Ai1a-2 in accordance with the MIGS recommendations** [38] **published by the Genome Standards Consortium** [39]

**MIGS ID**	**Property**	**Term**	**Evidence code**
	Classification	Domain *Bacteria*	TAS [40]
		Phylum *Proteobacteria*	TAS [41,42]
		Class *Alphaproteobacteria*	TAS [42,43]
		Order *Rhizobiales*	TAS [44]
		Family *Bradyrhizobiaceae*	TAS [45]
		Genus *Bradyrhizobium*	TAS [46]
		Species *Bradyrhizobium* sp.	IDA
	Gram stain	Negative	IDA
	Cell shape	Rod	IDA
	Motility	Motile	IDA
	Sporulation	Non-sporulating	NAS
	Temperature range	Unknown	NAS
	Optimum temperature	28°C	NAS
	pH range; Optimum	Unknown	NAS
	Carbon source	Varied	NAS
	Energy source	Chemoorganotroph	NAS
MIGS-6	Habitat	Soil, root nodule, host	TAS [6]
MIGS-6.3	Salinity	Non-halophile	NAS
MIGS-22	Oxygen requirement	Aerobic	NAS
MIGS-15	Biotic relationship	Free living, symbiotic	TAS [6]
MIGS-14	Pathogenicity	Non-pathogenic	NAS
	Biosafety level	1	TAS [47]
	Isolation	Root nodule of *Andira inermis*	TAS [6]
MIGS-4	Geographic location	Tres Piedras, Costa Rica	TAS [6]
MIGS-5	Sample collection	July 14, 2000	IDA
MIGS-4.1	Latitude	9.2835	IDA
MIGS-4.2	Longitude	−83.8533	IDA
MIGS-4.3	Depth	5 cm	IDA
MIGS-4.4	Altitude	50 m	IDA

Evidence codes – IDA: Inferred from Direct Assay; TAS: Traceable Author Statement (i.e., a direct report exists in the literature); NAS: Non-traceable Author Statement (i.e., not directly observed for the living, isolated sample, but based on a generally accepted property for the species, or anecdotal evidence). Evidence codes are from the Gene Ontology project [48,49].

### Symbiotaxonomy

Strain Ai1a.2 was isolated from the tree *Andira inermis* *,* Costa Rica [6]. The authentication of the symbiotic ability could not be performed using this host because seeds could not be accessed. The symbiotic capability of strain Ai1a.2 was tested on *Macroptilium atropurpureum* and this strain was able to nodulate this host. Acetylene reduction assays showed established nodules contained active nitrogenase, indicating an effective symbiosis with this host [6].

## Genome sequencing information

### Genome project history

This organism was selected for sequencing on the basis of its environmental and agricultural relevance to issues in global carbon cycling, alternative energy production, and biogeochemical importance, and is part of the Genomic Encyclopedia of Bacteria and Archaea, Root Nodulating Bacteria (GEBA-RNB) project at the U.S. Department of Energy, Joint Genome Institute (JGI). The genome project is deposited in the Genomes OnLine Database [20] and a high-quality permanent draft genome sequence in IMG [21]. Sequencing, finishing and annotation were performed by the JGI using state of the art sequencing technology [22]. A summary of the project information is shown in Table 2.

**Table 2:** **Project information**

**MIGS ID**	**Property**	**Term**
MIGS-31	Finishing quality	High-quality permanent draft
MIGS-28	Libraries used	Illumina Standard PE
MIGS-29	Sequencing platforms	Illumina HiSeq2000
MIGS-31.2	Fold coverage	Illumina, 119.7x
MIGS-30	Assemblers	Velvet version 1.1.04; Allpaths-LG version r42328
MIGS-32	Gene calling method	Prodigal 1.4
	Locus Tag	K288
	GenBank ID	AUEZ00000000
	GenBank release date	June 12, 2014
	GOLD ID	Gp0009887 [50]
	BIOPROJECT	195749
MIGS-13	Source Material Identifier	Ai1a-2
	Project relevance	Symbiotic nitrogen fixation, agriculture

### Growth conditions and genomic DNA preparation

*Bradyrhizobium* sp. Ai1a-2 was cultured to mid logarithmic phase in 60 ml of TY rich media on a gyratory shaker at 28°C [23]. DNA was isolated from the cells using a CTAB (Cetyl trimethyl ammonium bromide) bacterial genomic DNA isolation method [24].

### Genome sequencing and assembly

The draft genome of *Bradyrhizobium* sp. Ai1a–2 was generated at the DOE Joint Genome Institute (JGI) using the Illumina technology [25]. An Illumina standard shotgun library was constructed and sequenced using the Illumina HiSeq 2000 platform which generated 21,669,974 reads totaling 3,250.5 Mbp. All general aspects of library construction and sequencing were performed at the JGI and details can be found on the JGI website [26]. All raw Illumina sequence data was passed through DUK, a filtering program developed at JGI, which removes known Illumina sequencing and library preparation artifacts (Mingkun L, Copeland A, Han J, Unpublished). Following steps were then performed for assembly: (1) filtered Illumina reads were assembled using Velvet (version 1.1.04) [27], (2) 1–3 Kbp simulated paired end reads were created from Velvet contigs using wgsim [28], (3) Illumina reads were assembled with simulated read pairs using Allpaths–LG (version r42328) [29]. Parameters for assembly steps were: 1) Velvet (velveth: 63 –shortPaired and velvetg: −very_clean yes –exportFiltered yes –min_contig_lgth 500 –scaffolding no –cov_cutoff 10) 2) wgsim (−e 0 –1 100 –2 100 –r 0 –R 0 –X 0) 3) Allpaths–LG (PrepareAllpathsInputs: PHRED_64 = 1 PLOIDY = 1 FRAG_COVERAGE = 125 JUMP_COVERAGE = 25 LONG_JUMP_COV = 50, RunAllpathsLG: THREADS = 8 RUN = std_shredpairs TARGETS = standard VAPI_WARN_ONLY = True OVERWRITE = True). The final draft assembly contained 247 contigs in 246 scaffolds. The total size of the genome is 9.0 Mbp and the final assembly is based on 1,081.2 Mbp of Illumina data, which provides an average 119.7X coverage of the genome.

### Genome annotation

Genes were identified using Prodigal [30], as part of the DOE-JGI genome annotation pipeline [31,32]. The predicted CDSs were translated and used to search the National Center for Biotechnology Information (NCBI) non-redundant database, UniProt, TIGRFam, Pfam, KEGG, COG, and InterPro databases. The tRNAScanSE tool [33] was used to find tRNA genes, whereas ribosomal RNA genes were found by searches against models of the ribosomal RNA genes built from SILVA [34]. Other non–coding RNAs such as the RNA components of the protein secretion complex and the RNase P were identified by searching the genome for the corresponding Rfam profiles using INFERNAL [35]. Additional gene prediction analysis and manual functional annotation was performed within the Integrated Microbial Genomes-Expert Review (IMG-ER) system [36] developed by the Joint Genome Institute, Walnut Creek, CA, USA.

## Genome properties

The genome is 9,029,266 nucleotides with 62.56% GC content (Table 3) and comprised of 246 scaffolds. From a total of 8,584 genes, 8,482 were protein encoding and 102 RNA only encoding genes. The majority of genes (75.10%) were assigned a putative function whilst the remaining genes were annotated as hypothetical. The distribution of genes into COGs functional categories is presented in Table 4.

**Table 3:** **Genome statistics for** ***Bradyrhizobium*** **sp. Ai1a-2**

**Attribute**	**Value**	**% of total**
Genome size (bp)	9,029,266	100.00
DNA coding (bp)	7,683,922	85.10
DNA G + C (bp)	5,648,849	62.56
DNA scaffolds	246	100
Total genes	8,584	100.00
Protein coding genes	8,482	98.81
RNA genes	102	1.19
Pseudo genes	0	0.00
Genes in internal clusters	837	9.75
Genes with function prediction	6,447	75.10
Genes assigned to COGs	5,111	59.54
Genes with Pfam domains	6,590	76.77
Genes with signal peptides	837	9.75
Genes with transmembrane helices	1,914	22.30
CRISPR repeats	0	0.00

**Table 4:** **Number of genes associated with the general COG functional categories**

**Code**	**Value**	**% of total (5,698)**	**COG category**
J	185	3.25	Translation, ribosomal structure and biogenesis
A	0	0.00	RNA processing and modification
K	412	7.23	Transcription
L	223	3.91	Replication, recombination and repair
B	2	0.04	Chromatin structure and dynamics
D	33	0.58	Cell cycle control, cell division, chromosome partitioning
V	89	1.56	Defense mechanisms
T	234	4.11	Signal transduction mechanisms
M	277	4.86	Cell wall/membrane/envelope biogenesis
N	94	1.65	Cell motility
U	128	2.25	Intracellular trafficking, secretion, and vesicular transport
O	191	3.35	Posttranslational modification, protein turnover, chaperones
C	435	7.63	Energy production and conversion
G	340	5.97	Carbohydrate transport and metabolism
E	587	10.30	Amino acid transport and metabolism
F	77	1.35	Nucleotide transport and metabolism
H	198	3.47	Coenzyme transport and metabolism
I	311	5.46	Lipid transport and metabolism
P	364	6.39	Inorganic ion transport and metabolism
Q	256	4.49	Secondary metabolite biosynthesis, transport and catabolism
R	696	12.21	General function prediction only
S	566	9.93	Function unknown
-	3,473	40.46	Not in COGS

## Conclusions

*Bradyrhizobium* sp. Ai1a-2 is a member of a widely distributed *Bradyrhizobium* lineage, isolated from diverse legume hosts in North, Central and South America and South Africa. Little is currently known of the symbiotic associations of its host *Andira inermis*, apart from the discovery that the Puerto Rican isolate *Bradyrhizobium* sp. EC3.3 can also establish a symbiosis with this host [8]. The Costa Rican isolate Aia1-2 16S rRNA gene sequence is distinct to that of EC3.3 but identical to the 16S rRNA sequence of South African isolate *Bradyrhizobium* sp. WSM2783. Phylogentically, Ai1a-2 is closely related to *Bradyrhizobium* sp. Cp5.3 and *Bradyrhizobium* sp. Th.b2 from Panama and USA, respectively. The genome of *Bradyrhizobium* 1a-2 and Ai sp.WSM2783 were sequenced along with 23 other *Bradyrhizobium* genomes as a part of the GEBA-RNB project. Of these 25 sequenced strains, the *Bradyrhizobium* spp. Ai1a-2, WSM2783, Cp5.3, Th.b2 and *B. elkanii* USDA76T are affiliated with the *Bradyrhizobium elkanii* superclade. The *Bradyrhizobium* Ai1a-2 genome has the 2^nd^ lowest genome size (9 Mbp), gene count (8,584) and signal peptide percentage (9.75%) among these five strains. Comparing the genome attributes of *Bradyrhizobium* sp. Ai1a-2 along with other sequenced *Bradyrhizobium* genomes will be important for the understanding of the biogeography of *Bradyrhizobium* spp. interactions required for the successful establishments of effective symbioses with their diverse hosts*.*

## Additional file

Additional file 1:
**Associated MIGS record. Table S1.** Associated MIGS record for *Bradyhizobium* sp. Ai1a-2.

## Abbreviations

GEBA-RNB: Genomic Encyclopedia for Bacteria and Archaea-Root Nodule Bacteria
JGI: Joint Genome Institute
½LA: Half strength Lupin Agar
TY: Tryptone yeast
YMA: Yeast mannitol agar
CTAB: Cetyl trimethyl ammonium bromide
